# New insights into the role of the oral leukoplakia microenvironment in malignant transformation

**DOI:** 10.3389/froh.2024.1363052

**Published:** 2024-02-21

**Authors:** Wilfredo Alejandro González-Arriagada, Gisela Canedo-Marroquin, Daniela Adorno-Farías, Ricardo Fernández-Ramires

**Affiliations:** ^1^ Facultad de Odontología, Universidad de los Andes, Santiago, Chile; ^2^ Centro de Investigación e Innovación Biomédica, Universidad de los Andes, Santiago, Chile; ^3^ IMPACT-Center of Interventional Medicine for Precision and Advanced Cellular Therapy, Universidad de los Andes, Santiago, Chile; ^4^ School of Dentistry, Oral Medicine and Pathology Department, Universidad de Chile, Santiago, Chile; ^5^ Facultad de Medicina y Ciencias de la Salud, Universidad Mayor, Santiago, Chile; ^6^ Grupo Chileno de Cáncer Hereditario, Universidad de los Andes, Santiago, Chile

**Keywords:** oral cancer, oral leukoplakia, microenvironment, premalignant, lymphocyte, fibroblast, dysplasia, malignant transformation

## Abstract

Oral leukoplakia is the most frequent and potentially malignant lesion of the oral cavity. Although dysplasia grading remains the main factor for risk assessment, challenges persist in determining the exact risk of transformation, and the literature has focused on studying alternative biomarkers. The interaction between dysplastic epithelial cells and the microenvironment starts early, and the communication is mainly mediated by lymphocytes, inflammatory factors, fibroblasts, and the extracellular matrix, leading to dysplastic progression. Leukoplakia-infiltrating leukocytes (LILs) and leukoplakia-associated fibroblasts (LAFs) play crucial roles in the dysplastic microenvironment. The immune response is related to intraepithelial T lymphocyte infiltration, mechanisms of immunosuppression coordinated by regulatory T cells, M2 macrophage polarization, and increased numbers of Langerhans cells; in contrast, fibroblastic and extracellular matrix factors are associated with increased numbers of pro-tumorigenic myofibroblasts, increased expression of metalloproteinases vs. decreased expression of TIMPs, and increased expression of chemokines and other inflammatory mediators. The microenvironment offers insights into the progression of leukoplakia to carcinoma, and understanding the complexity of the oral microenvironment in potentially malignant diseases aids in determining the risk of malignant transformation and proposing new therapeutic alternatives.

## 1 Introduction

Oral leukoplakia is the most common potentially malignant lesion of the oral cavity, with a prevalence of 2%–3% of the global population; it is more frequent in patients older than 40 years and malignant transformation rates have been reported to range from 1% to 40%, with an average of 13% (1–3). While surgery remains the primary treatment option for isolated leukoplakia or proliferative verrucous leukoplakia with total excision of the lesion, approximately 80% of oral leukoplakia cases may be overtreated with surgery, resulting in unnecessary interventions (2, 4). Conversely, relying solely on follow-up strategies could result in delayed diagnosis and treatment in 10%–20% of cases, potentially impacting outcomes (5).

The histopathological grade of dysplasia has been used as the primary factor to determine the risk of malignant transformation of leukoplakia (6). The WHO three-tier system for grading oral dysplasia (mild, moderate, and severe) has been criticized, leading to proposals for a binary system (7). However, pathologists have encountered difficulties adopting the binary approach because it requires a more detailed analysis of qualitative and quantitative architectural and cytological criteria (8). This challenge, along with the risk of overlapping features and inter- and intra-observer variations, has led to the suggestion of using immunohistochemical biomarkers or artificial intelligence to improve leukoplakia categorization (9, 10).

Numerous biomarkers have been proposed with the potential to predict malignant transformation in oral leukoplakia. These markers focus on molecular alterations of dysplastic cells within the epithelium, such as proliferation or epithelial–mesenchymal transition markers, which are linked to the ability of the cell to modify its cytoskeleton or alter the expression of epithelial adhesion molecules on the surface (11, 12). Despite its potential significance, the study of the role of the subepithelial microenvironment in the malignant transformation of leukoplakia has been largely overlooked (13, 14). This minireview explores two key aspects: the inflammatory infiltrate, represented by the lymphocytes, which we will call leukoplakia-infiltrating leukocytes (LILs), and the leukoplakia-associated fibroblasts (LAFs).

## 2 Interaction between oral dysplasia and microenvironment

First, we need to understand how dysplastic epithelial cells initiate interactions with the subepithelial microenvironment. Changes initially manifest within the epithelium and are observable through histological features like hyperkeratosis (thickening of the keratin layer), acanthosis (increased cell layers), cell pleomorphism (various cell shapes and sizes), altered cytoplasm-to-nucleus ratio, atypical mitosis (irregular cell division), and nuclear pleomorphism (various nuclear shapes and sizes) (15). These cytological alterations may occur in the absence or with a minimal inflammatory response in the connective tissue. As dysplasia progresses, an inflammatory response may emerge even before the basement membrane is breached (16). This demonstrates the early interplay between the epithelium and the microenvironment, even in a pre-neoplastic state, strongly implicating a broader involvement of the subepithelial microenvironment in the malignant transformation process. Recently, a mathematical model proposed that the microenvironment plays a role in the epigenetic transition between healthy, dysplastic, and cancerous states of cells. This transition is stimulated by dysfunctional cells, creating positive microenvironmental feedback. This feedback loop has the potential to accelerate cancer initiation but also inhibit cancer progression (17).

To understand how this interaction unfolds, two possible hypotheses have been proposed. Despite a conserved basement membrane, the literature suggests an acellular mechanism, such as the direct communication between dysplastic cells and the underlying microenvironment through extracellular vesicles (EVs) or exosomes, which are capable of crossing the basement membrane (18), or soluble factors, such as cytokines, chemokines, and growth factors (19, 20). Alternatively, the other hypothesis involves the role of the immune system in inflammation through intraepithelial antigen-presenting cells such as Langerhans cells (LCs), which could take up antigens and present them to the immune system in the lymph nodes, ultimately triggering lymphocyte infiltration (21, 22). This inflammatory infiltrate needs to be distinguished from other non-specific inflammatory responses, such as those caused by chronic irritation in reactive hyperkeratosis related to mastication on an edentulous ridge or the use of dentures (23, 24). However, some studies suggest that even in mild leukoplakia, a gap in the basal membrane may occur. This microscopic damage, detectable only with a transmission electron microscope, may offer insights into the early interactions between dysplastic cells and the microenvironment (25). In potentially malignant colonic adenomatous polyps, an increase in CD3+ and CD8+ lymphocyte infiltration has been reported in lesions with high-grade dysplasia compared to those with low-grade dysplasia, suggesting early alterations in immune surveillance (26).

The molecular profile of oral leukoplakia with dysplasia reveals the downregulation of collagen synthesis pathways in the extracellular matrix (ECM) (27). This suggests a potential early role for altered stromal interactions in dysplastic development, possibly through reduced fibroblastic activity. Furthermore, studies have shown the reduced expression of type IV collagen (Col IV) and type VII collagen (Col VII) within the lamina densa of the basement membrane in oral leukoplakia with dysplasia (28, 29), a phenomenon strongly associated with upregulated expression and activity of metalloproteinase 2 (MMP-2) and metalloproteinase 9 (MMP-9), suggesting their potential role in mediating basement membrane degradation (29, 30), also related to reduced expression of TIMP metallopeptidase 1 (TIMP-1) (31). These observations suggest that dysplastic progression transcends the epithelial barrier, extending its molecular influence into the stroma through the basement membrane (32). Such findings may provide mechanistic insights into the initiation and progression of precancerous lesions, potentially leading to the development of prognostic markers and therapeutic targets.

Additionally, a high number of recurrent lesions have been reported in patients with oral leukoplakia (OL) (33). The development of these recurrences, even when surgical margins are histopathologically free of lesions, brings us to the concept of field cancerization. However, we believe it is important to consider whether a dysfunctional microenvironment, including lymphocytes and fibroblasts, also plays a role in this concept (34, 35).

## 3 Leukoplakia-infiltrating leukocytes (LILs)

The dynamic interplay between epithelial cells and their surrounding microenvironment is crucial for elucidating the early events in oral cancer development and identifying potential targets for intervention. In the context of oral epithelial dysplasia, immune cells play a critical role in shaping the microenvironment and influencing epithelial cell behavior. The stromal lymphocytic infiltrate in high-risk OL is likely to differ from that in non-progressive leukoplakia. The identification and characterization of this inflammatory population may help us understand how it contributes to early malignant transformation in the epithelium (36). The number of infiltrating lymphocytes was significantly elevated in cases of moderate and severe dysplasia compared with hyperkeratosis and mild dysplasia (37).

High lymphocyte infiltration in OL has been identified as a potential predictor of malignant transformation (38). While the presence of cytotoxic CD8+ T lymphocytes may indicate an attempt to eliminate dysplastic cells, other immune cell populations may contribute to an immunosuppressive environment, highlighting the duality of the immune system in both inhibiting and promoting tumor progression (39). The immunosuppressive environment is produced by the recruitment of regulatory T cells (Tregs) by dysplasia. Loss of SMAD-4 (38, 39), a known driver of tumorigenesis, is emerging as a promising biomarker for stratifying OL patients based on the risk of malignant transformation, as its reduced expression has been correlated with inflammatory stromal features, potentially creating an immunosuppressive microenvironment that hinders effective immune responses against dysplastic cells (40).

Deep learning algorithms have emerged as promising tools for predicting malignant transformation in oral epithelial dysplasia by analyzing peri-epithelial lymphocytic (PEL) activity. Trained on datasets containing both tissue architecture and individual cell features, architectural and cytological feature-specific models were built to accurately predict malignant transformation and recurrence. Based on peri-epithelial lymphocyte activity, this digital score aligns with existing knowledge that elevated PEL counts correlate with a higher risk of malignant transformation in oral leukoplakia, offering a potentially more precise and objective method for risk assessment and treatment decision-making (41).

The analysis of the CD4+/CD8+ lymphocyte ratio revealed no significant differences between different grades of dysplasia. However, the intraepithelial distribution of CD8+ lymphocytes demonstrated a highly significant difference between lichen planus and moderate-to-severe epithelial dysplasia. This distinct pattern suggests that CD8+ localization may offer a promising avenue for developing an adjunctive diagnostic tool to differentiate these entities. While further validation and larger-scale studies are needed, this finding holds the potential to improve diagnostic accuracy (36). In addition, it has been suggested that chronic inflammation in the esophagus or colon and bacterial infection, such as *Porphyromonas gingivalis*, in the oral cavity may promote the proliferation and survival of malignant cells by modulating the dysplasia-related immune response (42, 43).

Studies have shown that the presence of Tregs within the subepithelial stroma of leukoplakia is a potential indicator of a predisposition to carcinoma progression due to their immunosuppressive function (38). The expression of the PD1/PD-L1 axis, higher numbers of infiltrating lymphocytes, and the expression of Treg-related proteins (FOXP3, TGF-β, IL-6, and IL-10) may have a predictive potential role in malignant transformation. All of these proteins act as immune response regulators, but FOXP3 also has a role in immune tolerance, TGF-b is involved in tissue repair, and IL-6 and IL-10 are involved in inflammation (38, 44). It has been reported that FOXP3+ lymphocytes can be used to predict which OL may turn into cancer and that patients with OL showing high expression of FOXP3 should be monitored closely or undergo surgical excision (38).

Proliferative verrucous leukoplakia, a subtype with a high rate of malignant transformation, exhibited a distinct immune microenvironment compared to localized leukoplakia. CD8+ T cells and Tregs were more abundant in proliferative leukoplakia samples, often colocalizing at the dysplasia–stromal interface, with overexpression of programmed death-ligand 1 (PD-L1), a classic immune checkpoint related to the risk of malignant transformation (45, 46), suggesting that the PD-1/PD-L1 axis blockade can be used as immunotherapy in oral precancer (47). This suggests that CD8+ and Tregs may play a role in promoting malignant transformation, potentially serving as a prognostic marker for carcinoma risk. Recently, the role of tumor-infiltrating B cells has been associated with better progression, exerting an anti-tumor role and a positive synergistic effect on immunotherapy response, presenting antigens, and producing antibodies that target tumor cells (48). In OL, it was found that the proportion of B cells was higher in hyperkeratosis and mild dysplasia compared with severe dysplasia and OSCC (37).

The potential involvement of macrophages in the malignant transformation of oral leukoplakia has also been studied. A significant increase in macrophage infiltration and M2 polarization, which is an anti-inflammatory phenotype of macrophages, was observed in OL lesions with malignant transformation (49), and a synergistic activity was observed between Tregs and M2 macrophages to inhibit anti-tumor immune responses (50). This shift toward an M2 phenotype, characterized by pro-angiogenic, tissue remodeling, and immunosuppressive functions, suggests that M2 macrophages may actively contribute to cancer progression and the immunosuppressive environment (51). M1 macrophages, on the other hand, are typically associated with pro-inflammatory responses in host defense that are necessary for an efficient immune response to potentially malignant lesions (52). Additionally, studies have shown reciprocal signaling communication between basal layer cancer stem cells (CSCs) and stromal cells in dysplastic leukoplakia. CSCs can promote macrophage polarization toward pro-tumorigenic M2 phenotypes, which may contribute to malignant transformation and angiogenesis via VEGF secretion (53). This bidirectional interaction plays a crucial role in the progression of dysplastic leukoplakia to malignancy.

In oral epithelial dysplasia (OED), an oral pathology with the potential to become malignant, the number of Langerhans cell (LC) positively correlates with the severity of oral epithelial dysplasia (OED) lesions but exhibits a significant decrease in lesions with malignant transformation. This intriguing inverse relationship suggests that LCs play a complex role in OED progression (54, 55). Understanding their interactions with dysplastic cells, such as antigen presentation or immune suppression, is critical to unraveling the early events in oral cancer development and holds promise for improved diagnostic approaches.

Changes in some genes have been identified in oral epithelial dysplasia that may contribute to modifications in the tumor microenvironment of leukoplakia, such as in FAM198B (56). A positive correlation has been determined between FAM198B and the infiltration of major immune cells (including macrophages, Treg, and NK cells), and this gene has been suggested as a promising therapeutic target to reverse gastric cancer (57). Limitations in the reports analyzing M2 macrophages and Treg cells may be partly attributed to a potential bias in studies using tissue microarrays (58) instead of whole sections (50). The limited tissue representation in microarrays reduces the accuracy of the results, hindering our understanding of the specific roles of these cells in different cancers. Despite these limitations, substantial evidence supports the association between adverse clinical outcomes and M2 macrophage and Treg infiltration. M2 macrophages would promote Treg migration and upregulate chemokine receptors and adhesion molecules on these cells (58). Prognostic models derived from the study of such cells could predict the patient's sensitivity to immunotherapy and chemotherapy.

## 4 Leukoplakia-associated fibroblasts (LAFs)

The presence of myofibroblasts in OL and their potential contribution to malignant transformation remain subjects of ongoing debate. Some studies suggest a link between their presence and the severity of epithelial dysplasia, while others fail to find significant differences. It has been reported that a sparse and focal distribution of myofibroblasts was observed in a low percentage of OL samples, concluding that their presence did not significantly differ from normal oral mucosa (59–61). This finding challenges the notion of myofibroblasts as a reliable marker of malignant transformation in OL but also underscores the need for further investigation, given the heterogeneity of existing studies. Further studies using standardized methodologies and larger cohorts are crucial to definitively address the role of myofibroblasts in OL progression and their potential utility as diagnostic or prognostic markers (59). However, immunodetection of myofibroblasts does not aid in determining the malignant transformation potential of oral dysplasia. Myofibroblasts may contribute to oral tumorigenesis, and their density increases with the loss of cellular differentiation in OSCCs (61).

Furthermore, under culture conditions, dysplasia-associated fibroblasts exhibit increased MMP-9 expression and hepatocyte growth factor (HGF) secretion. This altered microenvironment affects the severity of dysplasia by inducing morphological changes in normal keratinocytes, highlighting the stromal influence on epithelial transformation (62). Studies have explored dysplastic cell–fibroblast interactions during early oral carcinogenesis. Notably, the dysplastic cells studied displayed a marker profile associated with the CSC phenotype (CD133+, CD44+, ALDH1A1, Notch1, Sox2), with expression levels positively correlating with dysplastic severity. Interestingly, neurogenic locus homolog protein 1 (Notch1) inhibition in these cells reduced cell proliferation and downregulated the CSC marker profile. Additionally, fibroblast-conditioned media have been shown to induce spheroid formation and proliferation in CSCs, suggesting a potential role for stromal factors in promoting their stemness and tumorigenic potential (63).

Oral leukoplakia-associated fibroblasts (LAFs) exhibit decreased secretion of chemokine (C-X3-C motif) ligand 1 (CX3CL1), an antifungal chemokine. Since *Candida albicans* is part of the oral microbiota, the OL microenvironment must deal with the imbalance with *C. albicans*. Thus, LAFs secrete CX3CL1 against *C. albicans* and contribute to the malignant transformation of OL. Chronic fungal infection has been linked to changes in the inflammatory microenvironment and immune dysregulation, both of which may promote the development of dysplastic lesions and malignant transformation (64). Notably, CX3CL1 was found to be significantly less abundant in LAFs compared to normal fibroblasts (NFs). Overexpression of CX3CL1 in LAFs significantly enhanced their antifungal defenses and reduced their susceptibility to *C. albicans*, suggesting a potential therapeutic strategy to improve immune responses and potentially halt leukoplakia progression (65).

Downregulated expression of transforming growth factor-β type II and III receptors (TβRII and TβRIII) is emerging as a potential early event in OL that may contribute to disease progression and aggressiveness. These receptors play a crucial role in TGF-β signaling, which regulates cell proliferation, differentiation, and apoptosis. Their loss due to the presence of TGF-β1 could lead to uncontrolled cell growth and immune evasion, promoting the development of aggressive OL lesions. Considering that high expression of CD44, a marker of CSCs, and TGF-β1 secretion, one of the most important cytokines in epithelial -mesenchymal transition (EMT), contribute to the malignant transformation of OL, their potential as therapeutic targets warrants further investigation. (66). Remarkably, LAFs exhibited significantly lower TβRII and TβRIII expression compared to normal oral fibroblasts (NFs), suggesting a potential link between stromal dysfunction and epithelial dysregulation in OL progression (67). These findings suggest that TβRII and TβRIII downregulation may serve as early biomarkers of OL aggressiveness and warrant further investigation as potential therapeutic targets to prevent disease progression.

## 5 Conclusions and future perspectives

**Figure 1:**
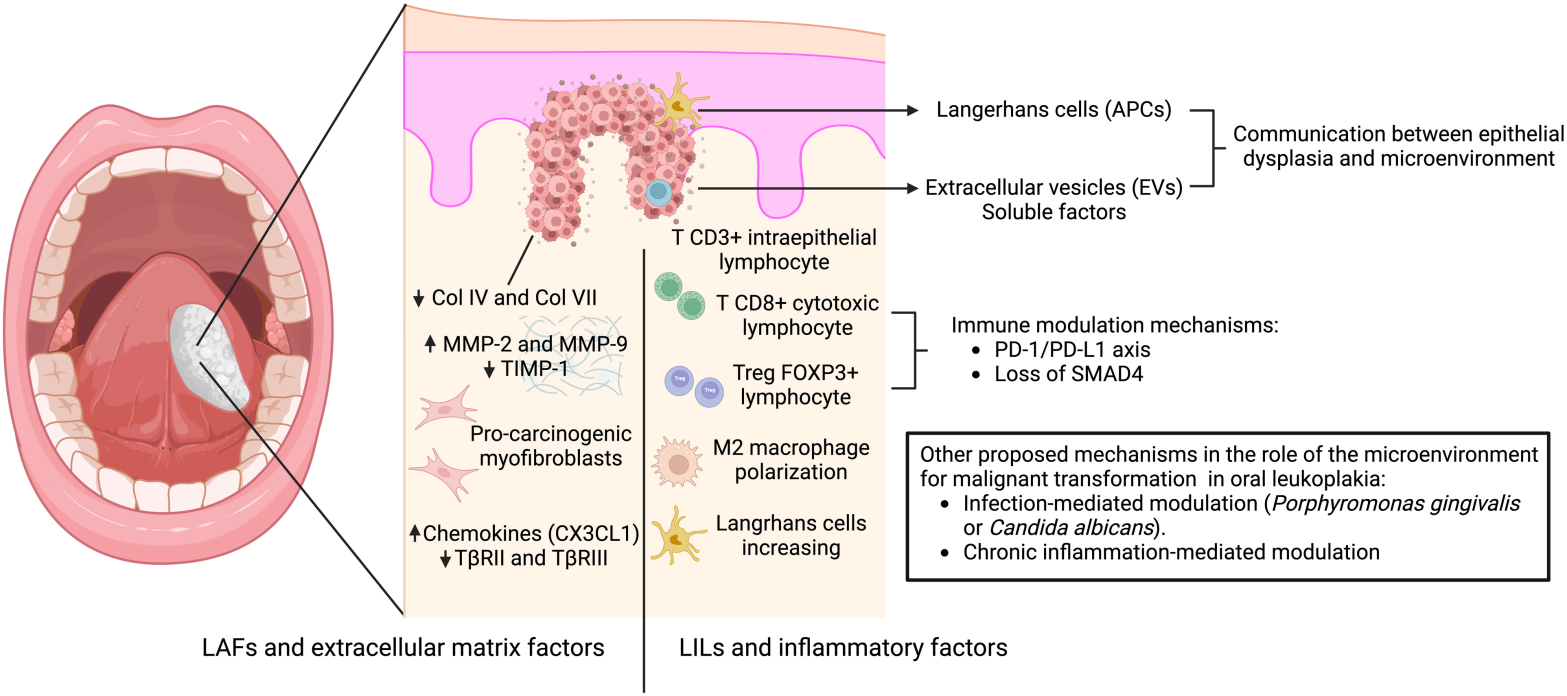
Mechanisms involved in the interaction between oral dysplastic epithelial cells and the microenvironment leading to malignant transformation. The proposed mechanism of early communication involves antigen-presenting cells (Langerhans cells), extracellular vesicles (EVs) and soluble factors, which promote the stromal response. This response is mediated by: (**A**) leukoplakia-infiltrating lymphocytes (LILs) and inflammatory factors related to intraepithelial T lymphocyte infiltration, mechanisms of immunosuppression coordinated by regulatory T cells through the PD1 -PDL1 axis, Treg-related proteins (FOXP3, TGF-β, IL-6, and IL-10), and loss of SMAD4 associated with M2 macrophage polarization and increased Langerhans cell numbers; and (**B**) leukoplakia-associated fibroblasts and extracellular matrix factors related to increased pro-tumorigenic myofibroblasts, decreased collagen expression in the basement membrane, increased expression of metalloproteinases vs. decreased TIMPs, and increased expression of chemokines vs. decreased receptors of TGF-β. Other mechanisms have been suggested to induce malignant transformation, including infection-mediated modulation (*Porphyromonas gingivalis* or *Candida albicans*) and chronic inflammation.

The microenvironment of potentially malignant oral diseases plays a crucial role in malignant transformation, and research in this field holds immense promise for future advancements in oral cancer prevention and treatment. This role is mainly related to two components (Figure 1):

a) Leukocytes and inflammatory factors: LILs in the microenvironment may influence cell basement membrane degradation and early invasion in the initial steps of carcinogenesis. Chronic inflammation, driven by factors like microorganisms or pro-inflammatory cytokines, can disrupt tissue homeostasis, leading to uncontrolled cell growth, and the higher number of Tregs lymphocytes promotes immune evasion.
b) Fibroblasts, extracellular matrix, and mechanical cues: The composition and stiffness of the extracellular matrix can influence epithelial cell behavior and adhesion, potentially favoring invasion and malignant transformation. LAFs, myofibroblasts, and other stromal cells within the underlying connective tissue may significantly influence the neoplastic transformation of oral leukoplakia. Dysregulation in these cells, such as reduced CX3CL1 secretion or TβRII/III downregulation, can create an environment that promotes dysplastic progression.

Future perspectives in potentially malignant oral diseases point to improved risk prediction of malignant transformation and manipulation of the microenvironment as a therapeutic strategy. The use of immune checkpoint inhibitors, specifically anti-PD-1 immunotherapy, has been studied for the treatment of oral leukoplakia, and recently, a clinical trial in oral high-grade epithelial dysplasia was reported with promising results (47).

The complexity and heterogeneity of the microenvironment of potentially malignant oral diseases pose a challenge in the development of universal diagnostic and therapeutic strategies; therefore, a better understanding of these processes in the microenvironment of potentially malignant lesions may help early detection and risk stratification with therapeutic strategies modulating the microenvironment with a personalized approach. Changes in the stroma may be an early manifestation of dysplastic development and malignant transformation. The study of these microenvironmental factors related to malignant transformation should be covered. Considering the difficulties of prognostic studies in oral leukoplakia, multi-institutional collaboration is needed to better characterize these cells and the mechanisms involved in the progression of leukoplakia to carcinoma.
